# Nutrient ingestion increased mTOR signaling, but not hVps34 activity in human skeletal muscle after sprint exercise

**DOI:** 10.1002/phy2.76

**Published:** 2013-09-23

**Authors:** Håkan C Rundqvist, Mats R Lilja, Olav Rooyackers, Krzysztofa Odrzywol, James T Murray, Mona Esbjörnsson, Eva Jansson

**Affiliations:** 1 Division of Clinical Physiology, Department of Laboratory Medicine, Karolinska InstitutetStockholm, Sweden; 2 Department of Clinical Physiology, Karolinska University HospitalStockholm, Sweden; 3 Division of Anesthesiology and Intensive Care, Department of Clinical Science, Intervention and Technology, Karolinska Institutet, Karolinska University HospitalStockholm, Sweden; 4 Centre for Cancer Research & Cell Biology, Queen's University BelfastCCRCB Building, Belfast, U.K; 5 Trinity Biomedical Sciences Institute, Trinity College DublinDublin, Ireland

**Keywords:** Amino acids, hormones, human, skeletal muscle, Western blot, Wingate test

## Abstract

Nutrient provision after sprint exercise enhances mammalian target of rapamycin (mTOR) signaling. One suggested that nutrient sensor is the class III phosphatidylinositol 3-kinase, vacuolar protein sorting 34 (Vps34), not previously studied in human skeletal muscle. It is hypothesized that oral ingestion of essential amino acids (EAA) and carbohydrates (Carb) increases Vps34 activity and mTOR signaling in human skeletal (hVps34) muscle after sprint exercise. Nine subjects were performed 3 × 30-sec all-out sprints with or without ingestion of EAA + Carb or placebo drinks in a randomized order with a month interval. Muscle biopsies were performed at rest and 140 min after last sprint and analyzed for p-mTOR, p-p70S6k, p-eEF2 and for hVps34 activity and hVps34 protein content. Venous blood samples were collected and analyzed for amino acids, glucose, lactate, and insulin. During the sprint exercise session, EAA, glucose, and insulin in blood increased significantly more in EAA + Carb than in placebo. P-mTOR and p-p70S6k were significantly increased above rest in EAA + Carb (*P* = 0.03, *P* = 0.007) 140 min after last sprint, but not in placebo. Activity and protein expression of hVps34 were not significantly changed from rest in EAA + Carb 140 min after the last sprint. However, hVps34 activity and protein expression tended to increase in placebo (both *P* = 0.08). In conclusion, on the contrary to the hypothesis, no increase in activation of hVps34 was found following sprint exercise in EAA + Carb condition. In spite of this, the results support an activation of mTOR during this condition. However, this does not exclude the permissive role of hVps34 in mediating the amino acid-induced activation of mTOR and muscle protein synthesis.

## Introduction

In sprint exercise, with great power development, there is a need for both large muscle mass and fast contracting muscles. Therefore, it is important to study the effect of sprint exercise on factors that regulate muscle protein metabolism and the possible additional effects of nutritional supplements. The few studies, that have been performed on this topic, show that there is low or no activation of the mammalian target of rapamycin (mTOR) and its downstream target p70S6k after sprint exercise, at least in males (Gibala et al. 2009; Esbjornsson et al. 2012b). mTOR and p70S6k regulate protein translation initiation and elongation as well as ribosomal biogenesis and their activation mostly indicates an increased protein synthesis (Kimball and Jefferson 2010) and muscle hypertrophy (Baar and Esser 1999; Bodine et al. 2001; Terzis et al. 2008), although this is not always the case (Greenhaff et al. 2008). Recently, Coffey et al. (2010) studied the influence of protein/carbohydrate provision on mTOR signaling and muscle protein synthesis after repeated bouts of 6-sec sprints. They found that the nutrient supplement enhanced both the downstream mTOR signaling and myofibrillar protein synthesis. The mechanism, by which the nutrients are sensed in the muscle cell, is poorly understood, although various signaling molecules have been suggested and studied in vitro. One of these suggestions is the class III phosphatidylinositol 3-kinase (PI3K), vacuolar protein sorting 34 (Vps34) (Byfield et al. 2005; Nobukuni et al. 2005).

Human Vps34 (hVps34) overexpression, in vitro has been shown to activate p70S6k, whereas an hVps34 knockdown was shown to block amino acid-induced activation of p70S6k (Nobukuni et al. 2005). Moreover, it was shown that addition of amino acids or glucose to starved nonmuscular cells leads to an increased activation of Vps34 and p70S6k (Byfield et al. 2005; Nobukuni et al. 2005). In addition, MacKenzie et al. (2009) demonstrated that leucine activated Vps34 in C2C12 myotubes and Gran and Cameron-Smith (2011) found that leucine increased the hVps34 protein concentration along with an increased p70S6k phosphorylation in human myotubes (Gran and Cameron-Smith 2011). These studies indicate that Vps34 contributes to the regulation of mTOR/p70S6k pathway by nutrients. However, most of these earlier studies were performed in vitro. Therefore, it is important to further explore the role of Vps34 as a nutrient sensor in vivo. Vps34 has previously been studied in rat skeletal muscle (Mackenzie et al. 2007; MacKenzie et al. 2009), but to the best of our knowledge, there are no earlier studies on Vps34 activity in human skeletal muscle tissue. It is hypothesized that oral ingestion of essential amino acids (EAA) combined with carbohydrates (Carb) increases the activity of hVps34 accompanied by increased phosphorylation of mTOR and p70S6k in human skeletal muscle after repeated bouts of 30-sec cycle sprint exercise.

## Methods

### Subjects

Nine healthy physically active subjects (eight males and one female) were recruited from the university area and sports/health clubs for the study. The inclusion criteria include: in good health, participation in leisure-time sports, but not at an elite level and age 20–35 years. The exclusion criteria were chronic disease, acute infection, severe asthma, or use of products containing nicotine. Health was checked by a general health questionnaire, followed by a 12-lead electrocardiogram registration. A questionnaire was used to estimate the physical activity level during leisure time. The subjects answered nine different questions from which an activity index (minimum value 5 and maximum value 20) was calculated (Jansson and Hedberg 1991). Fat-free body mass (FFM) was estimated from skin fold measurements in triceps, biceps, subscapula, and suprailiacal regions (Durnin and Womersley 1974). The maximal oxygen uptake (VO_2_ max) was determined using a standardized incremental cycle test to exhaustion on a cycle ergometer (Siemens-Elema, Solna, Sweden). Oxygen uptake was measured continuously utilizing a gas analyzer Vmax Encore System (VIASYS Healthcare Inc., Yorba Linda, CA). The subjects' physical characteristics are summarized in Table 1. All subjects were fully informed about the procedures and potential risks of the experiment before giving their written and verbal consent prior to participation. The study was approved by the Regional Ethical Review Board in Stockholm, Sweden.

**Table 1:** Physical characteristics in nine subjects

Physical characteristic	Mean ± SD
Age, year	28 ± 5
Height, cm	181 ± 10
Body mass, kg	77 ± 10
FM, %	15 ± 3
FFM, kg	66 ± 9
BMI, kg m^−2^	23.5 ± 2.1
VO_2_ max, L min^−1^	4.3 ± 0.6
Activity index, score	16 ± 3

FM, relative fat mass; FFM, fat-free body mass.

### Experimental protocol

**Figure 1:**
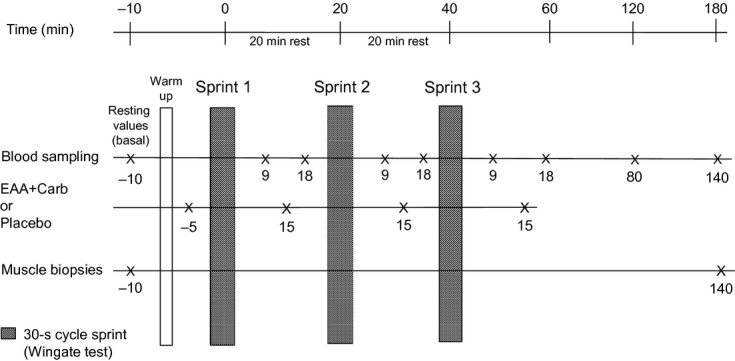
Schematic overview of the experimental protocol.

This randomized, single-blinded, placebo-controlled, crossover study is depicted in Figure 1. The sprint exercise protocol used in this study was designed to investigate the effects of 30-sec all-out sprints and the following recovery process. In order to repeat the 30-sec sprint with only a minimal loss of power or rate of muscle glycogenolysis in the second and third sprint as compared to the first sprint, a 20-min rest period separated the sprints.

All subjects repeated the sprint exercise protocol with or without nutrient ingestion with a month interval between occasions. The order of the experiments was randomized. On each of the two occasions, the subjects reported to the laboratory in the morning after an overnight fast. The subjects were instructed to ingest either an essential amino acid and maltodextrin drink (EAA + Carb) containing eight EAA, 300 mg × kg^−1^ body weight (Ajinomoto, Kanagawa, Japan) and maltodextrin, 1 g × kg^−1^ body weight (Faring AB Sports nutrition, Fast Carbs Natural, Järfälla, Sweden). Both EAA + Carb and placebo drinks were “Wild Berrie” flavored and contained salts and artificial sweetener, all to conceal the taste of the amino acids (Funlight, Wild Berries, Procordia Food AB, Eslöv, Sweden, diluted 1:4 in water, see also Table 2). Each subjects ingested a total volume of 12.5 mL kg^−1^ body weight of the drink delivered at four occasions (5 min before sprint 1 and 15 min after each sprint). The subjects performed the sprint exercise on a mechanically braked cycle ergometer (Cardionics, Bredäng, Sweden) according to the protocol in Figure 1 (Bar-Or 1987). After a brief warm up (1 min, with a braking load of 1 kg) the subjects were instructed to pedal as fast as possible without any resistance. When maximal pedaling speed was reached, an individual braking load set at 0.075 kp kg^−1^ body weight was applied to the flywheel, while the subjects continues to pedal “all out” for 30 sec. The subjects were performed in total three 30-sec sprint exercise bouts with 20-min rest between the sprints. A sensor-microprocessor assembly counted flywheel revolutions and the flywheel progression per pedal revolution was 6 m. Average power output was automatically printed every 5th second. Peak power (i.e., the highest 5-sec power) and mean power (the average power during the 30-sec sprint exercise) output was expressed in absolute values (W), relative to body mass (W × kg^−1^ body mass) and relative to FFM (W × kg^−1^ FFM), see Table 3. Power decrease was calculated as the difference between peak power and the last 5-sec power output divided by peak power. The subjects were in a supine position during the rest periods.

**Table 2:** Amino acid composition of the nutritional supplement drink

Essential amino acids	Weight%
Histidine	13.7
Isoleucine	9.4
Leucine	17.3
Lysine	18.0
Methionine	2.9
Phenylalanine	14.3
Threonine	13.7
Valine	10.7
Total%	100.0

300 mg kg^−1^ body weight of the essential amino acids and 1 g kg^−1^ body weight of maltodextrin were given to the subjects.

**Table 3:** Power performance in EAA + Carb and in placebo conditions in nine subjects

Performance	EAA + Carb	Placebo	*P-*value
Peak power, Watt (W)			
Sprint 1	855 ± 105	846 ± 113	ns
Sprint 2	843 ± 119	852 ± 111	ns
Sprint 3	839 ± 102	839 ± 109	ns
Mean power, W			
Sprint 1	626 ± 89	636 ± 92	ns
Sprint 2	633 ± 85	645 ± 90	ns
Sprint 3	613 ± 90	629 ± 84	ns
Peak power, W kg^−1^ body mass			
Sprint 1	11.2 ± 0.7	11.0 ± 0.7	ns
Sprint 2	11.3 ± 0.8	11.0 ± 0.7	ns
Sprint 3	10.9 ± 0.7	10.8 ± 0.7	ns
Mean power, W kg^−1^ body mass			
Sprint 1	8.1 ± 0.5	8.2 ± 0.6	ns
Sprint 2	8.2 ± 0.5	8.3 ± 0.4	ns
Sprint 3	8.1 ± 0.5	8.1 ± 0.5	ns
Peak power, W kg^−1^ fat-free mass			
Sprint 1	13.2 ± 1.2	12.9 ± 1.1	ns
Sprint 2	13.3 ± 1.4	13.0 ± 1.2	ns
Sprint 3	12.9 ± 1.2	12.8 ± 0.9	ns
Mean power, W kg^−1^ fat-free mass			
Sprint 1	9.6 ± 0.7	9.7 ± 0.9	ns
Sprint 2	9.7 ± 0.8	9.8 ± 0.7	ns
Sprint 3	9.3 ± 0.7	9.6 ± 0.7	ns

Values are the mean ± SD.

For the collection of blood samples, a Teflon catheter was inserted into an antecubital vein. Sampling occurred at 10 min before the first sprint, 9 and 18 min after each sprint and finally 80 and 140 min after the last sprint (Fig. 1). Muscle biopsy samples were taken from the lateral portion of the vastus lateralis muscle, ∼20 cm above the knee, under local anesthesia by applying the Bergstrom technique (Bergstrom 1975) from left or right leg before the sprints and from the contralateral leg 140 min after the last sprint. All muscle samples (50–100 mg) were quickly dissected free of any visible fat and connective tissue, and were frozen in isopentane cooled to its freezing point by liquid nitrogen. Muscle samples were stored at −80°C for later analyses.

### Blood preparation and analyses

Each blood sample (10 mL) was divided into two equal portions. One of these was transferred into sodium-heparinized tubes, and immediately centrifuged at 2000*g* (4°C) for 10 min. One-milliliter aliquots of the plasma were frozen in liquid nitrogen and stored at −80°C. The other portion of blood was transferred to a serum tube stored at room temperature for 20 min and then treated the same way as the heparinized blood. Plasma lactate concentration was analyzed using a Radiometer ABL 800 Flex blood gas analyzer (Berman & Beving Lab, Triolab, Gothenburg, Sweden). An electrochemiluminescence immunoassay was used to analyze serum insulin (Modular E170, Roche, Pharma, Stockholm, Sweden). Plasma glucose was analyzed enzymatically by a Beckman-Coulter (LX-20) instrument according to kit instructions. Briefly, amino acids were analyzed using high performance liquid chromatography as previously described by Vesali et al. (2002) using precolumn derivatization with orthophthaldialdehyde/3-mercaptopropionic acid (Alliance, Waters 2690, fluorescence detector Waters 474: Waters, Stockholm, Sweden).

### Muscle biopsy sample preparation and analyses

#### Western blot

Muscle samples (∼30 mg) were homogenized on ice using glass homogenizers in ice-cold buffer (20 μL mg^−1^ wet weight) containing 20 mmol L^−1^ 4-(2-hydroxyethyl)-1-piperazineethanesulfonic acid (pH 7.4), 1 mmol L^−1^ ethylene diamine tetraacetic acid (EDTA) (pH 7.4), 1 mmol L^−1^ Na_3_VO_4_, 5 mmol L^−1^ ethylene glycol tetraacetic acid (EGTA) (pH 7.4), 10 mmol L^−1^ MgCl_2_, 50 mmol L^−1^ β-glycerophosphate, 2 mmol L^−1^ dithiothreitol, 1% Triton X-100, and one tablet (per 10 mL) of Complete mini protease inhibitor tablets (Roche, Diagnostics, Indianapolis, IN) diluted in Milli Q® water (Millipore, Solna, Sweden). Homogenates were rotated (RM5 assistant 348, rotating mixer, Karl Hecht, Sondheim, Germany) for 60 min at 4°C and centrifuged at 15,000*g* for 10 min at 4°C to remove cell debris. The supernatants were collected and stored at −80°C. The protein concentration was determined using the Bio-Rad Bradford protein assay using a spectrophotometer (Molecular Device SpectraMax Plus 384, Sunnyvale, CA). The samples were diluted with homogenization buffer and Laemmli buffer (250 mmol L^−1^ Tris-HCl pH 6.8, 8% sodium dodecyl sulfate (SDS), 40% glycerol, 5% β-mercaptoethanol, and 0.002% bromophenol blue) to a final protein concentration of 2 μg μL^−1^ containing 25% Laemmli buffer. Following dilution, the samples were heated at 95°C for 5 min to denature proteins and stored at −20°C until further analysis.

Details of the Western blot procedures have been previously published (Apro and Blomstrand 2010) with slight modifications for this study. Samples containing total protein of 40 μg (30 μg in the case of anti-hVps34) were separated by SDS polyacrylamide gel electrophoresis (PAGE) on Criterion cell gels (Bio-rad lab, Hercules, CA) consisting of 7.5% acrylamide. Phosphorylated proteins were expressed relative to monoclonal anti-α-tubulin abundance to ensure equal protein loading. No significantly differences were appeared between pre- and postbiopsy sample for a-tubulin. Placebo and EAA + Carb samples from each subject were run on the same gel always beginning with a preexercise sample followed by the corresponding postexercise sample for each time point.

#### Antibodies

The primary antibodies (polyclonal) used were the following: phospho-mTOR (Ser2448; 1:1000; Cell Signaling Technology, Danvers, MA), phospho-p70S6k (Thr389; 1:1000; Cell Signaling Technology), phospho-eEF2 (Thr56; 1:2000; Cell Signaling Technology), hVps34 (1:300; Trinity Biomedical Science Institute, Trinity Collage Dublin, Ireland), and monoclonal α-tubulin (1:20,000; Sigma-Aldrich, St. Louis, MO). The secondary antibodies used were all from Cell Signaling Technology: anti-rabbit (1:10,000; p-mTOR, p-p70S6k, p-eEF2), anti-mouse (1:10,000; α-tubulin), and anti-sheep (1:10,000; anti-hVps34) IgG antibodies conjugated with horseradish peroxidase.

Due to the lack of biopsy material, two of the nine subjects in the study were not included in the Western blot analyses.

#### hVps34 activity assay

To produce protein extracts, muscle samples (∼30 mg) were powdered under liquid nitrogen using a mortar and pestle. Powdered frozen extracts were lysed using analysis buffer containing 50 mmol L^−1^ Tris-base pH 7.5, 1 mmol L^−1^ EGTA, 1 mmol L^−1^ EDTA, 1 mmol L^−1^ sodium orthovanadate, 10 mmol L^−1^ β-glycerophosphate, 50 mmol L^−1^ sodium fluoride, 5 mmol L^−1^ sodium pyrophosphate, 0.27 mol L^−1^ saccharose, 0.1% (v/v) β-mercaptoethanol and 0.3% (w/v) 3[(3-Cholamidopropyl)dimethylammonio]-propanesulfonic acid, and one tablet (per 10 mL) of Complete protease inhibitor (Roche, Diagnostics, Indianapolis, IN) to a final protein extract concentration of 2–5 mg muscle powder mL^−1^. The protein extract was agitated (Vibrax® orbital shaker, Sigma-Aldrich, Belfast, Ireland) at 1000 rpm, for 10 min at 4°C and centrifuged at 4°C for 15 min at 16,000*g*, to remove insoluble material. The protein concentration was determined using the DC protein assay (Bio-Rad) utilizing a spectrophotometer (Eppendorf® BioPhotometer, Hamburg, Germany).

Details of the hVps34 activity assay procedures have been previously published (MacKenzie et al. 2009) with slight modifications for this study. Samples were immunoprecipitated overnight at 4°C with 2 μg of sheep anti-hVps34 antibody (hVps34 antibodies, for activity measurements and Western blot analysis, were obtained from Dr. James Murray's laboratory at Trinity Biomedical Science Institute, Trinity Collage Dublin, Ireland) using a protein extract volume corresponding to 0.7–1.5 mg of total protein extract. hVps34 protein complexes were then immobilized on protein-G sepharose. Immunocomplexes were washed in Lysis buffer and in 60 μL TNE (10 mmol L^−1^ Tris-base, pH 7.5, 150 mmol L^−1^ NaCl, 1 mmol L^−1^ EDTA, 0.1 mmol L^−1^ Na_3_VO_4_), resuspended in TNE+ (TNE, 0.5 mmol L^−1^ EGTA, pH 8.0, 1: 1000 2-mercaptoethanol), and incubated with 20 μg hVps34 antigen peptide. Substrates for the assay were prepared by adding 10 μL of 100 mmol L^−1^ MnCl_2_ and 10 μL of 2 mg mL^−1^ phosphoinositol (PtdIns) (bovine liver, Avanti Polar Lipids, Alabaster, AL) to each sample. PtdIns was sonicated for 5 min prior to addition to the assay to generate micelles. Reactions were performed at 30°C with shaking throughout the assay and initiated with the addition of ATP mix (1 mmol L^−1^ unlabeled ATP, [*γ*-^32^P] ATP, H_2_O). After 10 min, reactions were terminated by the addition of 20 μL of 8 mol L^−1^ HCl and phase separated with 160 μL 1:1 chloroform: methanol and centrifuged for 1 min at 16,000*g*. The lower organic phase was spotted on an aluminum-backed silica TLC gel 60 F_254_ plate (Merck, Damstadt, Germany) and analyzed in a TLC chamber solvent system (60-mL chloroform, 47-mL methanol, 11.2-mL water, and 2-mL ammonium hydroxide). After the plates had developed sufficiently, they were air dried, wrapped in protective film, and exposed to a phosphorimager screen for 24 h before imaging with a phosphorimager (FLA-7000, Fujifilm, Tokyo, Japan). Incorporation of [*γ*-^32^P] ATP was quantified using Multi-Gauge software (Fujifilm).

The imprecision of the hVps34 activity measurements is not known for human skeletal tissue. Comparing samples obtained at rest (placebo compared to EAA), a coefficient of variation (CV) for the hVps34 activity of around 30% was found. However, this does not represents the imprecision of the method because a biological variation, over the 1 month separating the experiments, adds to the true and lower value of imprecision. The CV for the comparison of postexercise values was around 60%, indicating a greater variation after exercise, that is, in a situation where changes in hVps34 activity were expected to occur. This together with our control experiments in cell lines (see below) and the finding of the significant interaction between the two regression lines (changes in hVps34 activity vs. plasma glucose, one for each condition, see Fig. 7), indicates that the method has a potential to pick up changes in hVps34 activity in the actual experimental setup.

**Figure 2:**
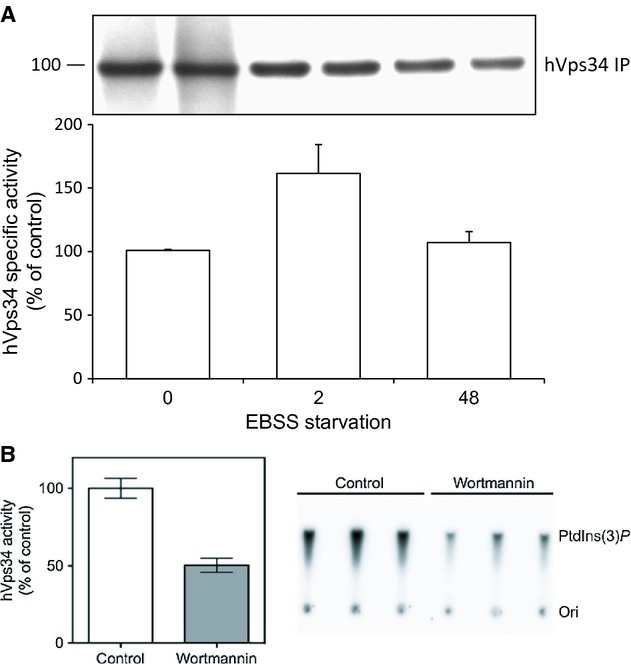
(A) Control experiments (*n* = 3) in MCF10a human breast carcinoma cell line following nutrient withdrawal (amino acids and glucose for 0, 2, and 48 h) show that an increase in hVps34 activity can be measured by the methodology used in this study to assess hVps34 activity in human muscle biopsies. EBSS, Earle's balanced salt solution. (B) In vitro analysis of hVps34 activity immunoprecipitated from HEK293 cells. HEK293 cells were grown to 80% confluence and treated with or without 100 nmol/L wortmannin for 1 h, prior to cell lysis. In each replicate, 1 mg of protein extract were subjected to overnight immunoprecipitation with 2 μg of anti-hVps34 antibody, prior to capture on Protein-G Sepharose beads. Enzymatic activity was measured as described in Methods and 32P-incorporation was quantified by densitometry and expressed as percent activity relative to control.

Control experiments in MCF10a, a human breast carcinoma cell line, following nutrient withdrawal show that an increase in hVps34 activity can be measured by the methodology used in this study to assess activity in human muscle biopsies, see Figure 2A. Another control experiment in HEK293, a human embryonic kidney cell line, shows that the activity of hVps34 can be inhibited by Wortmannin, see Figure 2B.

### Statistics

Values in the text are means ± SD unless stated otherwise. The *P-*values were accepted as statistically significant at *P* ≤ 0.05. A two-way analysis of variance (ANOVA) of repeated measures design was used to study the effect of condition (placebo or EAA + Carb) and time (alternatives depends on the variable). In the case of a significant interaction between condition and time, a one-way ANOVA of repeated measures design or Student's *t*-test for paired observations was used to evaluate changes by time separately for each condition. Student's *t*-test for group mean was used to evaluate difference between conditions at a few points of time.

The relationship between hvps34 activity and plasma glucose (area under the curve, AUC) was studied using linear regression model for each condition. To study possible interactions between condition and these regressions (differences in slope between conditions) a mixed model analysis with one within-group factor was performed with the independent variable plasma glucose as covariate.

## Results

### Sprint performance

The peak and mean power was not significantly changed on comparing sprint 1, 2, or 3 nor in placebo neither EAA condition (Table 3). Power decrease was approximately 45% irrespective of sprint 1, 2, or 3 in both conditions.

### Muscle protein data

#### Signaling response

**Figure 3:**
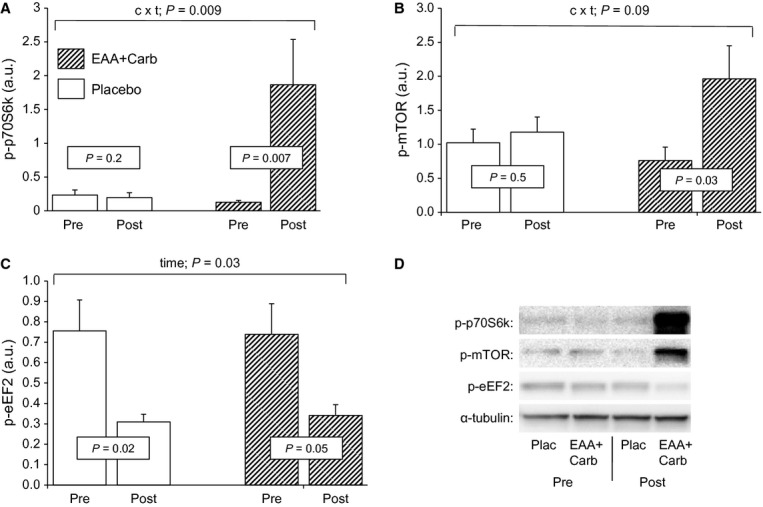
Phosphorylated (A) p70S6k (Thr389), (B) mTOR (Ser2448), and (C) eEF2 (Thr56) relative to α-tubulin at rest before the first sprint (pre) and 140 min after the last sprint (post) during EAA + Carb (white bars) and placebo conditions (hatched bars). Values are mean ± SE in arbitrary units of seven subjects; c, condition; *t*, time. (D). Representative immunoblots.

The phosphorylation of p70S6k at Thr389 was increased above rest (fold 14.7; *P* = 0.007) 140 min after sprint exercise in EAA + Carb condition, but not in placebo condition (condition x time; *P* = 0.009). A similar pattern was shown for the phosphorylation of mTOR at Ser2448, which also was increased above rest (fold 2.5; *P* = 0.03) 140 min after sprint exercise in the EAA + Carb condition but not in placebo (condition × time; *P* = 0.09). Phosphorylation of eEF2 at Thr56 was decreased below rest by ∼56%; (*P* = 0.03) 140 min after sprint exercise independent of condition (main effect of time; *P* = 0.03; Fig. 3).

#### hVps34 activity and protein expression

**Figure 4:**
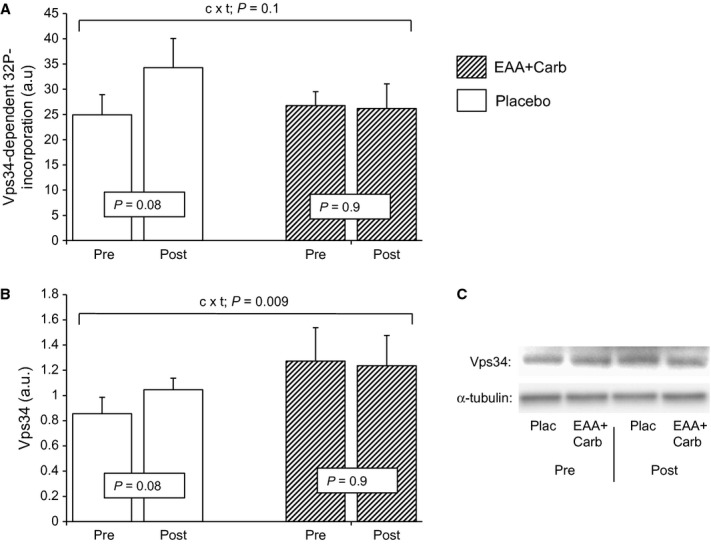
(A) The activity of hVps34 and (B) protein content of hVps34 relative to α-tubulin at rest before the first sprint (pre) and 140 min after the last sprint (post) in EAA + Carb (white bars) and placebo conditions (hatched bars). Values are mean ± SE in arbitrary units of nine and seven subjects, respectively. c, condition; *t*. time. (C). Representative immunoblots.

The activity of hVps34 was not significantly changed from rest in EAA + Carb condition 140 min after sprint exercise (Fig. 4A). However, an unexpected trend toward increased hVps34 activity (37%) was seen in the placebo condition (Fig. 4A; *P* = 0.08, condition × time: *P* = 0.1). A similar trend was seen for hVps34 protein expression (Fig. 4B).

### Blood data

Concentrations of plasma leucine, sum of EAA and of nonessential amino acids (NEAA), tryptophan, serum insulin, plasma glucose, and plasma lactate did not differ between conditions at rest (*P* > 0.05).

#### Plasma amino acids

**Figure 5:**
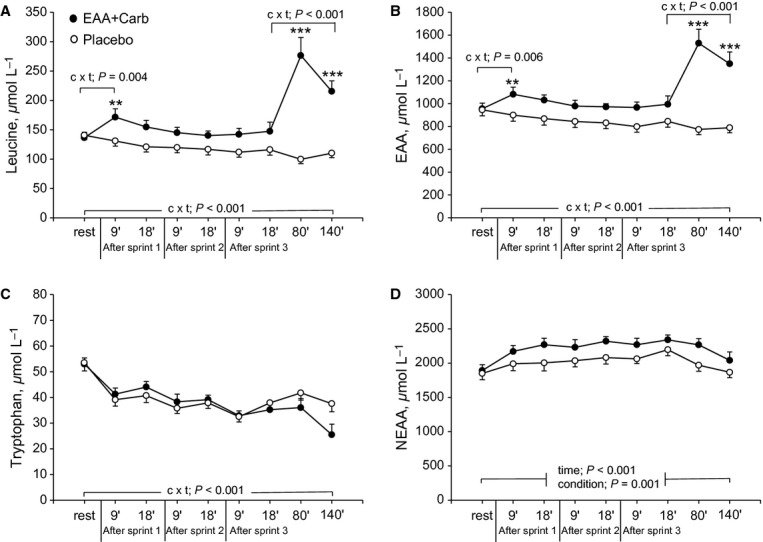
Concentration of (A) leucine, (B) EAA, (C) tryptophan, and (D) NEAA at rest and during the course of three bouts of sprint exercise with 20-min rest in between and 80 and 140 min after last sprint. EAA + Carb (closed symbols) and placebo conditions (open symbols). Values are mean ± SE (nine subjects). EAA denote essential amino acids and are the sum of histidine, isoleucine, leucine, lysine, methionine, phenylalanine, threonine, tryptophane, and valine. NEAA denote nonessential amino acids and are the sum of alanine, arginine, aspargine, glutamate, glutamine, serine, taurine, ornithine and glycine. Regarding the composition of the EAA + Carb drink see Table 2. c, condition; *t*, time. Significant difference between the conditions is denoted by **P < 0.01, ***P < 0.001.

The sprint exercise-induced changes by time in plasma leucine and EAA differed between the two conditions EAA + Carb and placebo, and were mainly localized to two periods, (Figs. 5A, B). The first one was identified between rest and 9 min after sprint 1. The second period appeared between 18 and 140 min after last sprint. In EAA + Carb condition, plasma leucine increased during the first period by 25% (*P* = 0.02, Fig. 5A) and during the second period by 88% (*P* = 0.001, Fig. 5A). In the placebo condition, plasma leucine decreased by approximately 20% as measured over the whole experimental period (rest to 140 min after last sprint, *P* < 0.001, Fig. 5A). At the peak level 80 min after the last sprint, the plasma leucine was 1.8-fold higher after EAA + Carb compared to placebo (*P* < 0.001, Fig 5A). Plasma EAA followed essentially the same pattern as plasma leucine (Fig 5B). Plasma tryptophan, which was not included in the nutrient drink, decreased over the experimental period in both conditions and more so in EAA + Carb than in placebo condition (53% vs. 30%, condition × time: *P* < 0.001, Fig. 5C). The NEAA did not change during the sprint exercise session, but were slightly higher in the EAA + Carb condition compared to the placebo condition, (condition: *P* < 0.001, Fig. 5D).

**Figure 6:**
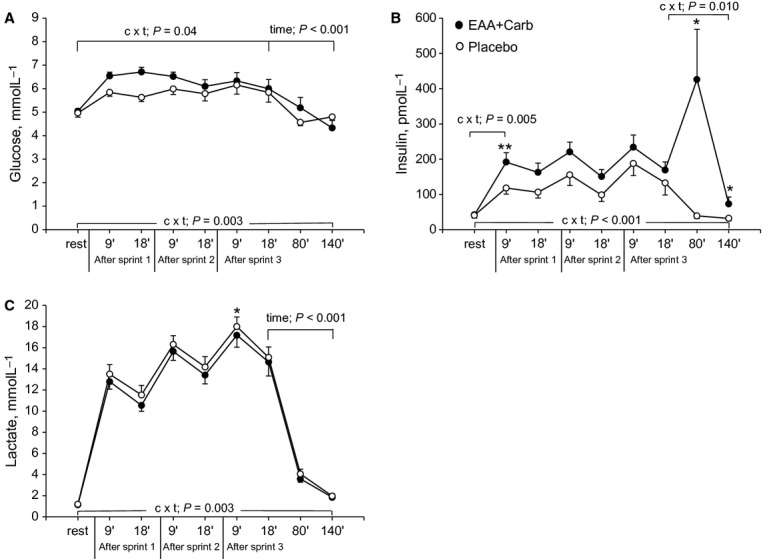
Concentration of (A) plasma glucose, (B) serum insulin, and (C) plasma lactate at rest and during the course of three bouts of sprint exercise with 20-min rest in between and 80 and 140 min after last sprint. EAA + Carb (closed symbols) and placebo conditions (open symbols). Values are mean ± SE of nine subjects. c, condition; *t*, time. Significant difference between the conditions is denoted by *P < 0.05, **P < 0.01.

*Plasma glucose* increased during the sprint exercise session and more so in EAA + Carb condition than in placebo (condition × time: *P* = 0.004). After the last sprint, plasma glucose decreased in both conditions by approximately 25% (time: *P* < 0.001, Fig. 6A).

*Serum insulin* increased nearly fourfold between rest and 9 min after sprint 1 in EAA + Carb condition, while, only half of as much in placebo (condition × time: *P* = 0.005). An accentuated increase was seen for serum insulin in EAA + Carb conditions from 18 min to 80 min after the last sprint (*P* = 0.08), while a decrease was seen in the placebo condition during this period (*P* = 0.01), leading to sevenfold higher concentration of serum insulin after EAA + Carb than placebo at 80 min after last sprint (*P* = 0.02). At 140 min after the last sprint, serum insulin was close to basal resting levels in both conditions, (Fig. 6B).

*Plasma lactate* increased markedly during the sprint exercise session in both conditions (time: *P* < 0.001). However, the peak level at 9 min after the last sprint 3 was 5% lower in EAA + Carb compared to placebo (condition: *P* = 0.03). Plasma lactate decreased similarly in both condition from 18 min to 140 min after last sprint, (time: *P* < 0.001, Fig. 6C).

### Correlations

**Figure 7:**
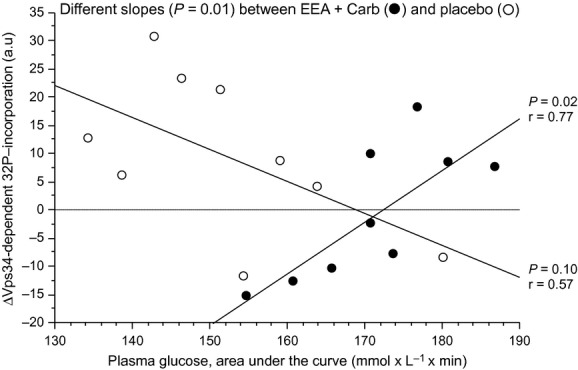
The relationship between the sprint exercise-induced increase in hVps34 activity and plasma glucose estimated by area under the curve (before sprint 1–9 min after sprint 2) in nine subjects. Note the opposite slopes when comparing the two conditions.

The increase in hVps34 activity was inversely related to the plasma glucose level expressed as AUC in placebo condition, but directly related to plasma glucose in EAA + Carb condition (*P* = 0.1 and *P* = 0.02, respectively). These two regression lines had significantly different slopes (*P* = 0.01). This indicates that in placebo condition, the increase in hVps34 activity was greater the lower plasma glucose concentration, while in EAA + Carb the increase in hVps34 activity was greater the higher plasma glucose concentration. The five subjects with the lowest plasma glucose levels (AUC) during placebo condition showed a significant increase in Vps34 activity after the sprint exercise (*P* = 0.013). Plasma leucine was higher in EAA + Carb condition as compared to placebo, which may add to explain this difference between conditions regarding the relationship between hVps34 and plasma glucose. A similar pattern was seen if AUC was calculated for extended time up to 18 min after the last sprint, but not if the whole area from rest to 80 or 140 min after the last sprint was included (Fig. 7).

## Discussion

The major and novel finding in this study was that the measured hVps34 activity did not increase in human skeletal muscle after sprint exercise combined with oral ingestion of EAA and Carb (EAA + Carb). In spite of this, there was a marked increase both in plasma EAA and p70S6k Thr389 phosphorylation in skeletal during the current experimental setup. This is, to the best of our knowledge, the first report of hVps34 activity measurements in human skeletal muscle.

The hypothesis in this study was based on in vitro experiments, showing that Vps34 is activated by amino acids or glucose (Byfield et al. 2005; Nobukuni et al. 2005; MacKenzie et al. 2009). No previous studies have been conducted in vivo on the effects of exercise alone or in combination with nutrient ingestion on Vps34 activity in human skeletal muscle. However, there are some in vivo studies on Vps34 protein measurements in skeletal muscle related to nutrition in nonhuman species (Suryawan and Davis 2010). In skeletal muscle of fed mice, there was lower detectable Vps34 protein expression along with reduced abundance of Vps34-beclin 1 complexes as compared to the fasted state (Yamada et al. 2012). These data were supported by an earlier in vitro study by Tassa et al. (2003), who demonstrated that amino acid-fed C2C12 myotubes demonstrated a reduced class III PI3K (likely Vps34) activity and reduced abundance of class III PI3K–beclin 1 complex.

The lack of consistency between the various studies discussed above might be explained by two distinct roles of Vps34. One is to control growth by activating mTOR when amino acids are present. Whereas a second role is to induce autophagy during amino acid-deprived conditions. These opposing roles of Vps34 are thought to be possible by the formation spatially distinct Vps34 containing complexes mediating either mTOR activation and cell growth or autophagy (Burman and Ktistakis 2010; Ktistakis et al. 2012).

Contrary to our hypothesis, the hVps34 activity and protein concentration tended to increase (*P* = 0.08) after sprint exercise in the nutrient poor (fasted) condition in this study, that is, the placebo condition. The novel interaction reported in this study, between increased hVps34 activity and plasma glucose levels in the EAA + Carb and placebo conditions, emphasizes the complexity of hVps34 activation and regulation (Fig. 6). In fact, it supports the model of the dual functions of this enzyme, as described by Burman and Ktistakis (2010), where Vps34 activity is stimulated by both nutrient-rich and nutrient-poor conditions. The lack of statistical significance for the increase in Vps34 activity in the placebo condition might be a question of experimental statistical power. However, it may also depend on a true interindividual variation in exercise-induced changes in hVps34 activity related to the degree of lack of nutrition in the placebo condition: subjects with the lowest plasma glucose level in the placebo condition consistently showed an increase in hVps34 activity, whereas those with higher plasma glucose did not (Fig. 7).

An increase in hVps34 activity in a nutrient-poor condition is in line with the finding by Mackenzie et al. (2007); MacKenzie et al. (2009), who found an increased Vps34 activity after exercise in the fasted rat. These are the only publications on Vps34 activity in skeletal muscle after exercise in any organism. As mentioned earlier, Vps34 is essential for autophagy, a process by which cells degrade cytosolic components (e.g., damaged organelles and unused long-lived proteins) by forming a double-walled vesicular structure, called an autophagosome, which fuses with lysosomes, thereby delivering cargo for destruction and recycling. This process is thought to help to maintain a balance between degradation, synthesis, and subsequent recycling of cellular products (Burman and Ktistakis 2010; Ktistakis et al. 2012). The formation of autophagosomes is initiated by hVps34 complexes that minimally contain hVps15 and beclin 1 (Mizushima 2007; Burman and Ktistakis 2010; Ktistakis et al. 2012). Autophagy is not only induced changes activated by nutrient deficiency (Mizushima 2007), but also by exercise conditions such as hypoxia and oxidative stress (Azad et al. 2009; Noman et al. 2012). Interestingly, exercise was found to induce autophagy in skeletal muscle of mouse (Grumati et al. 2011; Nair and Klionsky 2011). Moreover, He et al. (2012) demonstrated that the beneficial effects of exercise were dependent on an intact autophagy phenotype.

Several studies have demonstrated that phosphorylation of p70S6k is increased after resistance exercise and that this increase is potentiated by nutrients such as EAA (Dreyer et al. 2008; Apro and Blomstrand 2010). The nutrient-induced changes in mTOR signaling after sprint exercise is less well studied. However, this study of 30-sec sprints and the earlier study by Coffey et al. (2010) of 6-sec sprints show that nutrients have similar effects on mTOR activation after sprint and resistance exercise. In addition, the eEF2 phosphorylation, known to activate translational elongation, decreased to a similar extent after nutrient ingestion and placebo, despite the marked nutrient-induced increase in p70S6k phosphorylation. This indicates a potentiated activation of translation initiation, but not of translational elongation during the nutrient ingestion condition. A similar finding has been presented previously, after resistance exercise (Dreyer et al. 2008). Irrespective of the lack of a measurable increase in hVps34 activity after sprint exercise combined with nutrient ingestion, a pronounced increase in the phosphorylation of p70S6k was found. Alternatively, p70S6k could be activated via the insulin–Akt–mTOR signaling pathway (Hillier et al. 2000; Greenhaff et al. 2008). Ingestion of amino acids is known to increase plasma insulin levels (Floyd et al. 1966). In fact, in this study, a higher serum insulin concentration was found during EAA + Carb condition and the peak concentration occurred simultaneously with the peak for plasma EAA. This suggests that an EAA-induced increase in serum insulin may activate p70S6k through the Akt–mTOR pathway. In fact, Coffey et al. (2010) showed that Akt phosphorylation was increased after EAA ingestion in parallel with increased p70S6k phosphorylation. Additional evidence comes from a study by Anthony et al. (2002), who demonstrated in rat skeletal muscle, that a leucine-induced increase in p70S6k phosphorylation was dependent on insulin (Anthony et al. 2002).

Most earlier studies of sprint training have been performed without control of nutrient supply pre- and post the training sessions and there are no clear cut results regarding sprint training-related changes in, for instance, muscle mass (Esbjörnsson et al. 1993; Ross and Leveritt 2001). Therefore, it would be of great interest to study the effect of nutrients on sprint training-induced changes in muscle mass. The finding in this study of a decrease in plasma EAA – during repeated bouts of 30-sec sprint exercise (Esbjornsson et al. 2012a) in fasting condition, support the need for nutrient supply after sprint exercise to maintain or increase muscle mass.

One limitation of this study is that only one postexercise biopsy was taken approximately 2 h after the last bout of exercise. This was justified by a desire to avoid a residual effect of a preceding biopsy on protein activation. We cannot exclude the possibility of an earlier, transient increase in hVps34 activity following the last bout of exercise, related to the increasing plasma EAA level. However, the plasma EAA levels were still clearly elevated at the time of the postexercise biopsy obtained 140 min after the last sprint, suggesting activation conditions for hVps34. Further studies are necessary to explore the possibility of transient hVps34 activation. Another limitation of the study is that we did not had enough biopsy material to analyze other potential activators of mTOR following sprint exercise combined with nutrient ingestion such as membrane-associated amino acid transporters (Drummond et al. 2011; Suryawan and Davis 2011), Ser/Threonine protein kinase (MAP4K3 [Findlay et al. 2007]) or Rag GTPases (Han et al. 2012).

In conclusion, this is the first report demonstrating hVps34 activity in human skeletal muscle. Our data demonstrate that measurements of lipid kinase function and hVps34 specificity are possible in human subjects and allow for further studies on this important aspect of mTOR signaling. On the contrary to the hypothesis, no increase in activation of hVps34 was found following sprint exercise in a nutrient-enhanced condition. In spite of this, the results strongly support an activation of mTOR during this condition. However, this does not exclude the permissive role of hVps34 in mediating the amino acid-induced activation of mTOR and muscle protein synthesis. Even though not hypothesized we propose that an activation of hVps34 may occur following sprint exercise in the fasted condition (placebo) as earlier demonstrated after skeletal muscle contractions in rat (MacKenzie et al. 2009).
